# Response surface optimization of a vortex-assisted dispersive liquid–liquid microextraction method for highly sensitive determination of repaglinide in environmental water by HPLC/UV

**DOI:** 10.1186/s13065-022-00826-w

**Published:** 2022-05-14

**Authors:** Amira H. Kamal, Mohamed A. Hammad, Reham E. Kannouma, Fotouh R. Mansour

**Affiliations:** 1 Department of Pharmaceutical Analytical Chemistry, Faculty of Pharmacy, Tanta University, Tanta, 31111 Egypt; 2 Department of Analytical Chemistry, Faculty of Pharmacy, University of Sadat City, Tanta, 32958 Egypt; 3 Pharmaceutical Services Center, Faculty of Pharmacy, Tanta University, Elgeish Street, Tanta, 31111 Egypt

**Keywords:** Repaglinide, Nateglinide, Vortex-assisted DLLME, HPLC/UV, Chemometrics, Response Surface Optimization

## Abstract

A vortex-assisted dispersive liquid–liquid microextraction (DLLME) method, mated to chemometrics and combined with HPLC/UV detection was optimized and validated for enrichment and determination of repaglinide in environmental samples using nateglinide as an internal standard (IS). A phosphate buffer (10 mM, pH 2.5): acetonitrile (45:55, v/v) was used as a mobile phase with a flow rate of 1 mL/min in an isocratic elution mode. Chemometrics-assisted optimization was performed using a quadratic integrated d-optimal design. The developed model assessed the statistical significance of the independent variables and their interactions to attain the optimum conditions revealing that extractant type, extractant volume and pH are the most influential factors. Optimization of the extraction procedures was performed with the aid of Design Expert 8® software, which suggested 58 different experiments. The optimal conditions were 30 µL of 1-octanol as extractant, 100 µL of acetonitrile as a disperser at pH 8. Under the optimized conditions, the method showed linearity over the range of 1–100 ng/mL with a limit of detection of 0.4 ng/mL. The accuracy, the intra- and inter-day precision were assessed, the %recoveries were found to be between 98.48 and 100.81% with %RSD lower than 1.3. Using chemometrics in method optimization helped achieve the maximum possible enrichment with the least effort, time, and reagents while considering all possible interactions between variables.

**Supplementary Information:**

The online version contains supplementary material available at 10.1186/s13065-022-00826-w.

## Introduction

**Fig. 1:**
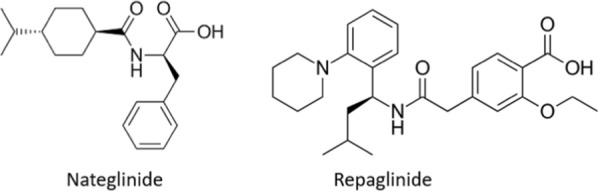
Chemical structure of repaglinide and nateglinide (IS)

Non-insulin-dependent diabetes mellitus (NIDDM) is a chronic disease characterized by a defect in insulin secretion or insulin resistance. The Meglitinide family, including repaglinide and nateglinide (Fig. 1), can stimulate insulin release from the β-cells of the pancreas by inhibiting potassium ion-dependent ATP channels [1, 2]. Repaglinide is rapidly absorbed after oral administration of a 2 mg tablet achieving a C_max_ of 28 ng/mL within half an hour [1]. It is eliminated via biliary-faecal and urinary excretion within 96 h after administration [3].

Great attention has been paid to the life and behaviour of pharmaceuticals in water cycles. Many pharmaceuticals are taken up easily by the human body to exert their activity. Based on the pharmacology of the drug, it will be excreted unchanged or as a mixture of metabolites in urine or faeces reaching the wastewater treatment plants [4]. These drugs may retain in the sludge or may be metabolized to a more hydrophilic form passing through the wastewater treatment plant and reaching the receiving waters. Levels of many drugs are hardly reduced and so, they can be detected in wastewater-treatment plant effluents. Pharmaceuticals may have adverse effects on aquatic organisms even if they are present at low concentrations [5].

Because antidiabetic drugs such as repaglinide are used for the treatment of chronic disease, they are continuously released to the surface water systems through industrial and sewage treatment plants at very low concentrations. Several studies have been reported for quantitation of repaglinide, using highly sophisticated, expensive, and sensitive techniques [6–11] due to the low concentrations of repaglinide in aqueous samples. Sample preparation and preconcentration are important to analyze the low concentrations of repaglinide. Solid-phase extraction and liquid phase extraction are the main common sample preparation techniques [12, 13]. Compared with other sample preparation techniques, dispersive liquid–liquid microextraction is widely used because it is rapid, simple, inexpensive, and eco-friendly. Dispersive liquid–liquid microextraction is a ternary solvent system in which only microliters of organic solvent as an extractant in addition to a disperser solvent are added to the aqueous sample to form a cloudy state [14]. Deep eutectic solvents could be used in dispersive liquid–liquid microextraction [15, 16]. The dispersion process improves the surface area contact between both the extractant and the aqueous sample and hence attains the equilibrium rapidly [17]. Several mechanical powers can assist the disperser to improve the dispersion process. Manual shaking is considered the simplest one, but it rarely achieves an effective dispersion and always needs to be combined with other dispersion methods. It results in poor stability of the dispersion and as a result a contradiction of the analysis results between the samples using the vortex mixing improves the dispersion and its uniformity [18]. Using the vortex mixing or other mechanical powers enhances the extraction efficiency and reaches equilibrium more rapidly [19].

Optimization of the experimental variables of vortex-assisted DLLME is critical to get the best conditions that give an acceptable response at the lowest possible concentration of the analyte. One-factor-at-a-time (OFAT) approach is used by most of the studies of analytical methods by studying the effect of each variable individually while keeping other variables constant. This approach consumes a lot of time, effort, and reagents. Moreover, it may not consider interactions between variables, which may affect the optimization results [20]. Recently, chemometric-assisted optimization of analytical methods has provided a theoretical basis for the processes and provides mathematical models that evaluate the independent parameters’ significance and interactions. This approach saves time, reagents and experimental work, extracts the more significant variables as well as determines what combination of the levels of variables achieve the best method performance [21–23].

In this paper, a vortex-assisted dispersive liquid–liquid microextraction (DLLME) method combined with HPLC/UV detection was optimized and validated for preconcentration and quantitation of repaglinide in an aqueous environmental sample (river water and tap water). The experimental variables that affect the method were evaluated and optimized with the aid of chemometrics design of experiment (DOE) using a quadratic integrated D-optimal design to achieve the best performance with the least time and effort. The method was compared with other reported methods and was found simpler, easier, faster, more sensitive than the reported HPLC/UV methods and comparable in performance to LC/MS without the need for such a sophisticated technique or experienced operators.

## Experimental

### Apparatus and software

A Cyan-CL008benchtop centrifuge from Cypress Diagnostics (Langdorp, Belgium) was used for phase separation. The pH measurements were carried out using a Jenway 3510 pH-meter of Cole-Parmer (Saint Neots, UK). A vortex (2800 rpm) (London, UK) was used for sample dispersion. Optimization of the extraction procedures was performed with the aid of Stat-Ease’s Design Expert 8® software (Minneapolis, MN, USA). A Dionex UltiMate 3000 HPLC of Thermo Scientific™ (Sunnyvale, CA, USA) was used. The HPLC instrument included an LPG-3400SD quaternary pump, a WPS-3000TSL autosampler, a TCC-3000SD column thermostat, and a VWD-3000 variable wavelength detector. The HPLC was controlled by a computer using Chromeleon 7 software for data acquisition and processing.

### Reagents and standards

Repaglinide (99.8%) and nateglinide (99.2%) were kindly supplied by Sigma Pharmaceutical Industries (Quwaysna, Egypt). Methanol (HPLC grade), acetonitrile (HPLC grade) and potassium dihydrogen phosphate were purchased from Merck (Darmstadt, Germany). 1-Octanol, 1-decanol, 1-undecanol and 1-dodecanol (analytical grade) were purchased from Sigma Aldrich (Darmstadt, Germany). Glycerol, propylene glycol, sodium hydroxide, and phosphoric acid were purchased from Piochem (6th of October, Egypt). Distilled water was used for the preparation of aqueous solutions. Drug-free human plasma from healthy volunteers (Blood Bank, Benha University Hospitals, Benha, Egypt) was used for the application of the vortex-assisted DLLME method. The plasma samples, stored at − 20 °C were thawed to room temperature before use.

### Chromatographic conditions

A Thermo Hypersil® ODS C18 column (150 mm × 4.6 mm, 5 µm) was used as the stationary phase at 30 ℃. A phosphate buffer (10 mM, pH 2.5): acetonitrile (45:55, v/v) was used as a mobile phase with a flow rate of 1 mL/min in an isocratic elution mode. Detection was carried out at 210 nm with a run time of 10 min and an injection volume of 3µL.

### Standard solutions and sample pretreatment

Standard stock solutions of repaglinide and nateglinide (internal standard) were prepared in methanol (0.5 and 1 mg/mL, respectively) and stored at 4 °C. A standard solution of repaglinide was prepared by diluting the stock solution with deionized water to the concentration of 1 µg/mL. Environmental water samples were collected from the Nile River (Egypt) and lab tap water, and stored in amber glass bottles at 4 °C. No filtration was applied to environmental water before microextraction. Environmental water samples were treated with a standard phosphoric acid solution or a standard sodium hydroxide solution to adjust the pH. Further dilutions were prepared by spiking the pretreated environmental water with repaglinide and nateglinide to obtain the working solutions of the desired concentrations.

### Vortex-assisted DLLME procedures

A volume of 10 mL of environmental water (pH 8) spiked with repaglinide and nateglinide was mixed with 30 µL of 1-octanol (extractant) and 100 µL of acetonitrile (disperser) in a 15 mL screw cap plastic tube. The mixture was vortexed for 1 min for dispersion, then the sample was centrifuged for 5 min at 1792 G-force for phase separation. The upper 1-octanol layer was withdrawn using a 25µL Hamilton syringe, collected in a micro vial, and injected (3 µL) into the HPLC column. Vortex-assisted DLLME procedures were performed at room temperature (25 °C).

### Calculation of enrichment factor (EF)

Several parameters can be used to evaluate the performance of the vortex assisted-DLLME method. The enrichment factor (EF) was used to assess the effect of experimental conditions on the efficiency of vortex assisted-DLLME and was calculated from the following equation:

$$EF= \frac{{\text{C}}_{\text{org}}}{{\text{C}}_{\text{aq}}}$$

where *C*_*org*_ and *C*_*aq*_ are the concentration of repaglinide in the organic phase and the initial concentration of repaglinide in the aqueous sample, respectively [24].

### Analytical performance of the developed method

Validation of the vortex-assisted DLLME HPLC/UV method according to ICH guidelines was done by spiking tap water samples with different concentration levels of repaglinide and a constant level of nateglinide. The calibration curve was constructed by plotting peak area ratios (repaglinide-to-nateglinide) versus concentrations of repaglinide (n = 6). Limits of detection (LOD) and quantitation (LOQ) were used for evaluation of the method sensitivity through determining the lowest concentration producing signal-to-noise ratio of 3 and 10, respectively. The selectivity of the method was evaluated by checking the chromatograms of blank samples for any interfering peaks at the same retention time of repaglinide. The selectivity was further confirmed by making minor modifications in the mobile phase composition (Acetonitrile:buffer ratio) and checking the chromatograms for the appearance of any unexpected peaks. The intra- and inter-day accuracy and precision (n = 3) were studied using spiked environmental water samples with repaglinide at concentrations of 10, 40, 70 ng/mL using 1000 ng/mL nateglinide as internal standard (IS). The accuracy and precision were expressed as % recovery and % RSD, respectively.

## Results and discussion

DLLME has been extensively used for the preparation of various samples with different matrices including environmental water [25], soil [26], food [27], plasma [28, 29], serum [30], urine and saliva [31]. DLLME has been applied for determinations of pharmaceuticals [17], pesticides [32], insecticides [33], toxicants [34], and natural products [35]. In this work, a dispersive liquid–liquid microextraction methodology was combined with HPLC/UV detection for preconcentration and determination of repaglinide in environmental water samples. The continuous consumption of antidiabetic drugs increases the possibility of its presence in industrial and municipal sewage water. Repaglinide cannot be detected in aqueous samples without pretreatment due to its low concentrations. Vortex-assisted DLLME efficiency was affected by experimental conditions including extractant type and volume, disperser type and volume, and pH of the sample [36]. To attain the utmost enrichment of repaglinide, it was essential to optimize these variables. This optimization was performed on two sequential steps: studying the effects of different experimental variables to set the limits, and optimizing these conditions by simultaneous changes within the specified limits. The mentioned chromatographic conditions showed retention times of 8.3 and 5.5 min for repaglinide and nateglinide, respectively.

### Selection of extractant type and volumes

Suitable extractants should have certain characteristics to achieve the desired purpose of use. These characteristics include high safety, environmental benignness, low miscibility with the aqueous phase, the ability to extract the target analyte, and compatibility with the analytical instrument [37]. High density halogenated hydrocarbon solvents such as chloroform and dichloromethane usually were used but they are highly toxic [38]. Other low-density solvents such as long-chain alcohols are suitable alternative extractants [39]. Based on preliminary experiments, it was found that long-chain alcohols such as 1-octanol, 1-decanol, 1-undecanol, and 1-dodecanol were good extractants of repaglinide (Additional file 1: Fig. S1) so, they were chosen to be studied using experimental design optimization. The volume of extractant was also critical for the enrichment of the analyte. Increasing the extractant volume may lead to decreasing the enrichment factor due to the dilution effect [40]. Decreasing the volume beyond 30 µL lead to decreasing the available volume for analysis [38] so, volumes ranging from 30 to 150 µL were chosen for the subsequent optimization.

### Selection of disperser type and volumes

The main requirement of a suitable disperser is its miscibility with both the extractant solvent and aqueous phase to ensure efficient contact between both of them [41]. Introducing new dispersers like glycerol and propylene glycol enhances the green aspects of analysis. Based on preliminary experiments, methanol, acetonitrile, glycerol, and propylene glycol showed reasonable extraction efficiency (Additional file 1: Fig. S2), while acetone and ethanol were excluded as they showed overlapping peaks at the same retention time of the analyte. This could be due to the presence of impurities in acetone and ethanol. Volumes ranging from 100 to 500 µL were chosen for subsequent optimization. Volumes lower than 100 µL and higher than 500 µL were tried but they showed low enrichment factors (Additional file 1: Fig. S3). Low volumes of disperser are not enough to disperse the extractant in the aqueous phase while high volumes may enhance the miscibility of the extractant in the aqueous phase making the phase separation difficult [36] or it may be due to increasing the solubility of repaglinide in water which minimizing the extraction efficiency [24].

### Selection of pH range

Ionizable drugs are greatly affected by pH, hence optimization of pH during vortex-assisted DLLME procedures is critical. The extraction of the drug is affected by the degree of ionization which in turn depends on pH. The best pH is the one that ensures that most of the drug molecules are in the unionized form with sufficient hydrophobicity to be more extractable in the organic phase [40, 41]. Repaglinide has two pK_a_ values of 6.20 (basic) and 3.96 (acidic) for the amine and the carboxylate groups, respectively [42]. So, no specific pH within the HPLC working range could be selected to make the two functional groups of repaglinide in the unionized forms. For this reason, a wide range of pH values from 2 to 8 was chosen for further optimization.

### Optimization of extraction procedures

Method optimization in DLLME could be performed using either the classical one factor at a time approach [43] or chemometrics methods including response surface methodology [44]. In response surface methodology, the different experimental variables are studied simultaneously which saves time, effort and consider potential interactions between variables. Accordingly, the results of the preliminary study were used as a base for further optimization of vortex-assisted DLLME procedures of repaglinide using response surface methodology, with aid of Design Expert 8® StatEase software. The model studied the interaction between the different variables: type of extractant, the volume of extractant, type of disperser, volume of the disperser, pH of the sample. The developed model decreased the time required to reach the optimum conditions in comparison with the one factor at a time approach. Besides, the model helped to predict enrichment factors at any given condition and the critical experimental variables.

The five variables (extractant type, extractant volume, disperser type, disperser volume, and sample pH) were varied over a program-suggested 58 runs using a quadratic integrated D-optimal design (Additional file 1: Table S1). The levels of each variable were chosen based on the previous results, as shown in Table 1. The desirability criteria were set to select the conditions that achieve the maximum response, where the latter was defined as the EF of each run. The desirability function attempts to reach operating conditions that guarantee compliance with the criteria of the involved response. The *d* values could be between 0 and 1, where 0 indicated undesirable conditions while 1 could hypothetically be attained at fully desirable conditions [21, 45].

**Table 1:** The levels of variables used in the optimization process

Variable	Range
Type of extractant	1-octanol, 1-decanol, 1-undecanol, 1-dodecanol
Volume of extractant	30–150 µL
Type of disperser	methanol, acetonitrile, glycerol, propylene glycol
Volume of disperser	100–500 µL
Sample pH	2 –8

**Fig. 2:**
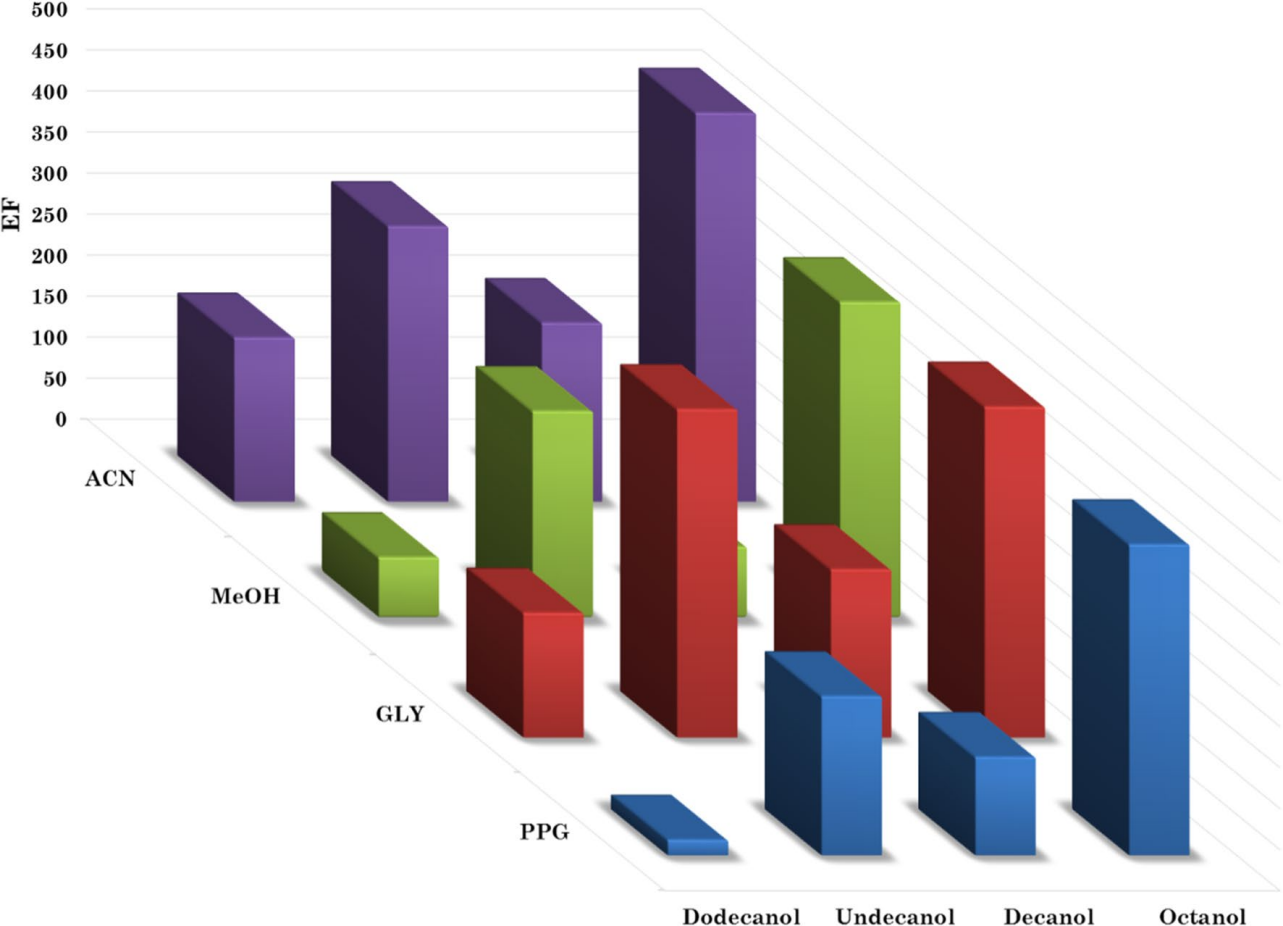
The effect of interaction between the type of extractant and the type of disperser on the enrichment factor. ACN: acetonitrile, MeOH: methanol, GLY: glycerol, PPG: propylene glycol

The enrichment factors (EF) of different extractant/disperser combinations were calculated by the developed model, as shown in Fig. 2. This simultaneous optimization considered the possible interactions between variables and help reach the actual optimum. The results revealed that the best combination consisted of 1-octanol as an extractant with acetonitrile as a disperser. It also showed that 1-octanol was always the best extractant, no matter which disperser was used. It is worth mentioning that glycerol showed comparable results with methanol, which highlights the potential use of glycerol as a green disperser. Based on the optimization results, three variables had substantial effects on the EFs. These variables included the extractant type, the extractant volume, and the pH, while the other two variables (the disperser type and volume) had observably lower effects on EFs (Additional file 1: Fig. S4).

**Fig. 3:**
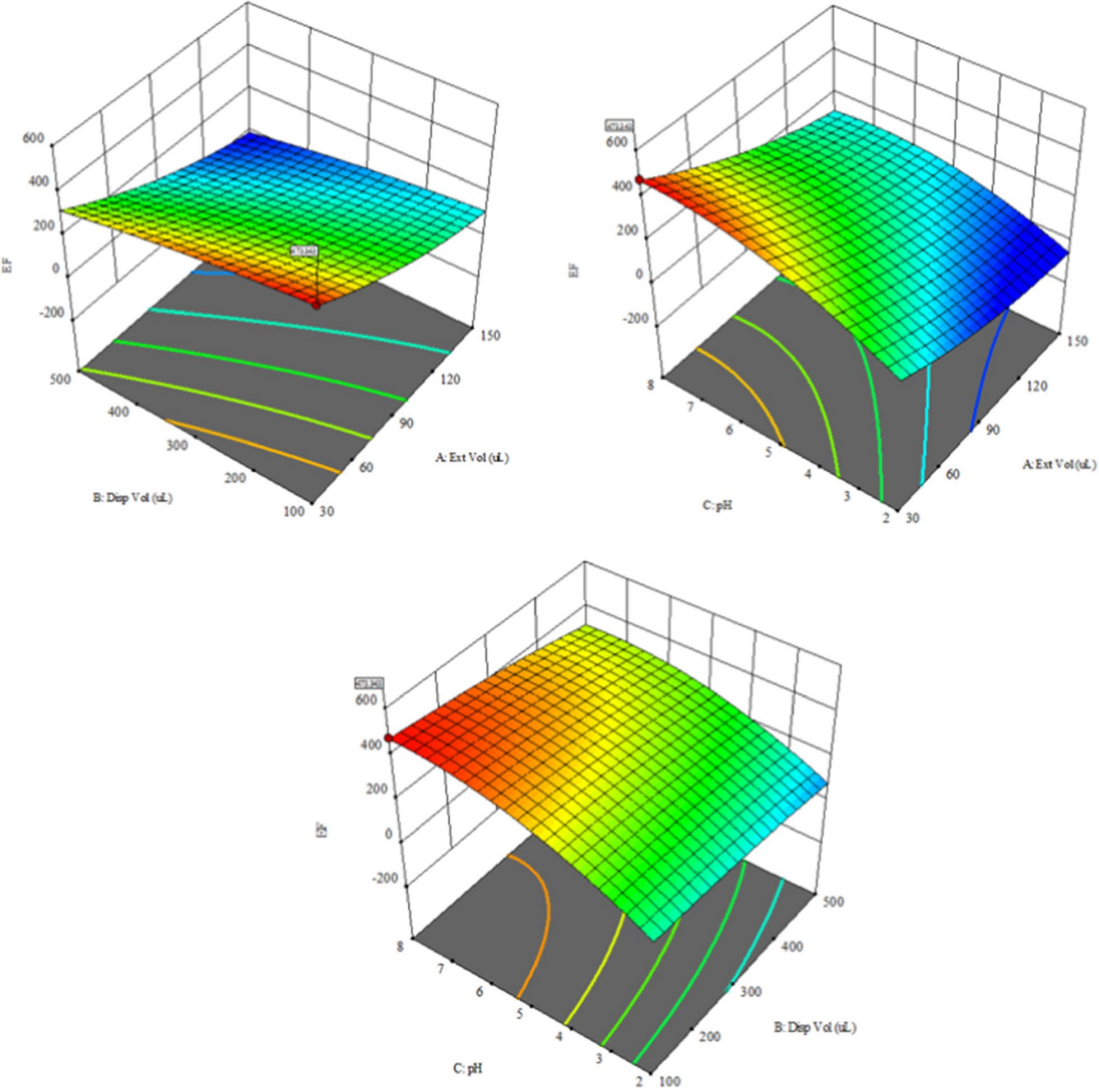
Response curves of the optimized conditions for the experimental variables of extractant volume (µL), disperser volume (µL), pH

As expected, the EFs were inversely proportional to the extractant volumes due to the possible dilution effect, with the maximum EF achieved using 30μL. Increasing the disperser volumes decreased the EFs due to the expected increase in the volume of extractant/disperser mixture after phase separation. Using pH 8 led to the greatest enrichment factor at which the amine functional group (basic pK_a_ = 6.20) of repaglinide would be in the unionized form, which was preferably extractable. As shown in Fig. 3, the highest *d* value was attained under the experimental conditions of 30 µL of 1-octanol (extractant) and 100 µL of acetonitrile (disperser) at pH 8.

The model was evaluated by the fraction of design space (FDS) that could give an estimate of the response with enough precision. FDS needs to be higher than 80% for the model to be considered reliable [46]. FDS was estimated by the software (Additional file 1: Fig. S5) and was found to be 88.2% with a standard error of 0.978 at a 95% confidence level. The model predictability was tested by plotting the actual EFs versus the predicted EFs (Additional file 1: Fig. S6), satisfactory results were obtained. The Model had an F-value of 4.38, which implied that the model was significant. The P-values (0.0031) was less than 0.0500, which indicated that the model terms were significant, with only a 0.31% chance that an F-value this large could occur due to noise.

**Fig. 4:**
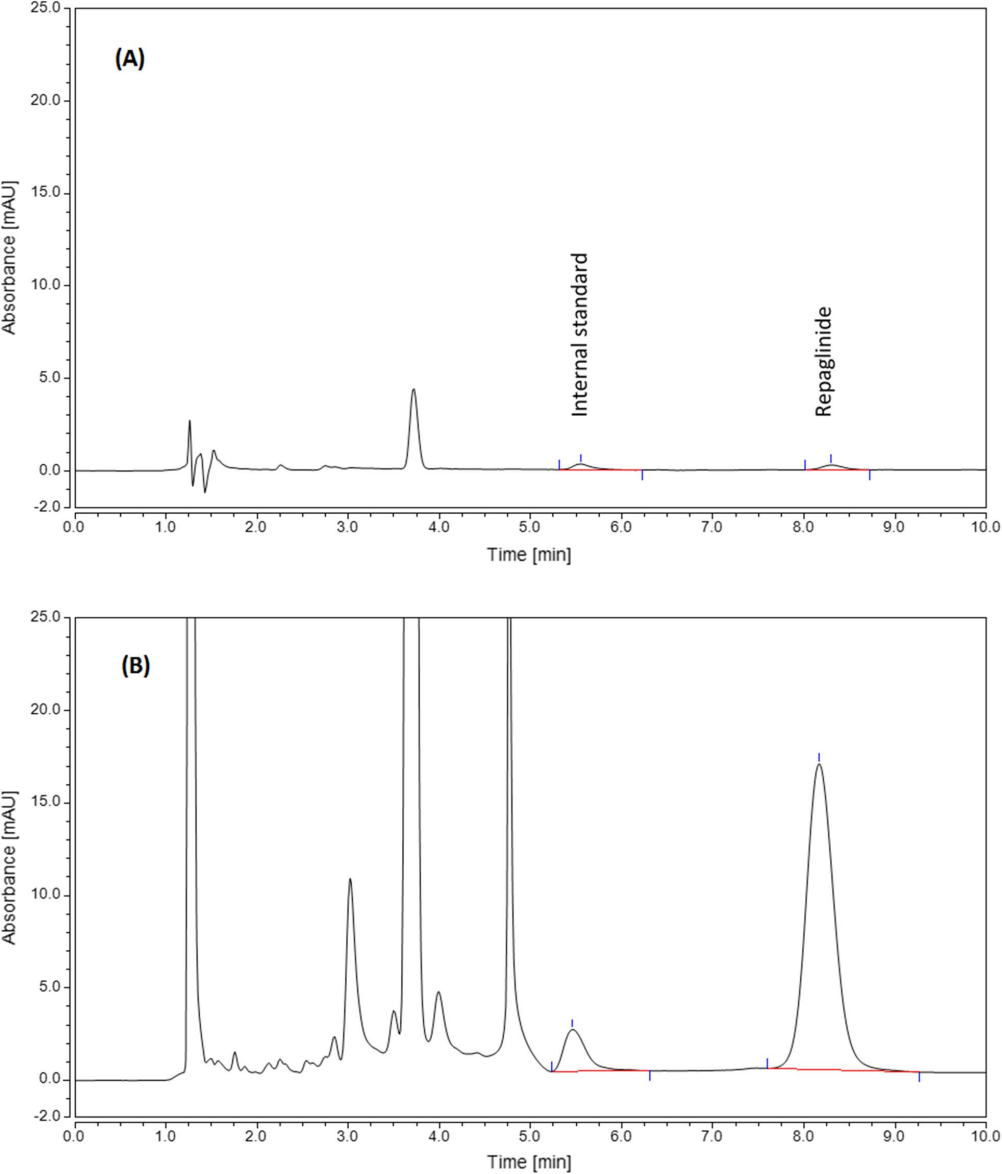
Chromatogram of repaglinide **A** before and **B** after vortex assisted-DLLME. Microextraction conditions: extractant type: octanol, extractant volume: 30μL, disperser type: acetonitrile, disperser volume: 100 μL, vortex time: 1 min, centrifugation time: 5 min, diluent: pH = 8

The optimum conditions were tried to extract repaglinide from environmental waters using vortex-assisted DLLME and were compared with the untreated aqueous samples. Nateglinide was used as an internal standard to improve the method’s accuracy and precision. Figure 4 shows the chromatographic separation of repaglinide before and after vortex-assisted DLLME using the optimum condition. The samples treated with vortex-assisted DLLME were markedly concentrated with an EF of 480.

### Analytical performance of the vortex-assisted DLLME HPLC/UV method

Under the optimized conditions, the developed vortex-assisted DLLME HPLC/UV method was validated for linearity, specificity, limits of detection and quantitation, intra- and inter-day accuracy and precision using spiked samples. The method showed good linearity in the range of 1–100 ng/mL with a correlation coefficient of (r) of 0.9969 (n = 6). The LOD was found to be 0.4 ng/mL with a signal-to-noise ratio of 3 (Table 2) while the LOQ was found to be 1 ng/mL with a signal-to-noise ratio of 10. The selectivity of the method was shown by the absence of any peaks in the same retention time of repaglinide in blank chromatograms and chromatograms of other chromatographic conditions. The method reliability was assessed via using control charts of repaglinide retention times and areas under curves of the internal standard. As shown in Additional file 1: Fig. S7, the results showed that none of the reported data exceeded the control limits and more than 95% of the data were below the warning limits. The % recovery (found concentration/ spiked concentration × 100) was used for the evaluation of accuracy while the precision was evaluated by %RSD. The intra-day accuracy and precision (n = 3) were found to be 99.46% and 1.20%RSD while inter-day accuracy and precision (n = 3) were found to be 99.57% and 0.13% RSD (Table 3).

**Table 2:** Regression parameters for determination of repaglinide by the proposed method

Analyte	Linear regression					LDR (ng/mL)	LOD (ng/mL)	EF
	Slope	SE of slope	intercept	SE of intercept	r value			
Repaglinide	0.0370	0.0009	0.1436	0.0571	0.9969	1–100	0.4	480

SE: standard error; r: correlation coefficient; LDR: linear dynamic range; EF: enrichment factor

**Table 3:** Intra and inter-day accuracy and precision (n = 3) for determination of repaglinide by the proposed method

Parameter	Accuracy and precision					
	Intra-day			Inter-day		
	Spiked conc. (ng/mL)	Found conc. (ng/mL)	%Recovery	Spiked conc. (ng/mL)	Found conc. (ng/mL)	%Recovery
	10	9.9	99.1	10	9.9	99.7
	40	39.4	98.5	40	39.8	99.5
	70	70.6	100.8	70	69.7	99.5
Mean			99.5			99.6
% RSD			1.2			0.13

%RSD: Percent relative standard deviation

### Applications

The validated developed vortex-assisted DLLME method was applied for the determination of repaglinide in environmental water using the optimum conditions. River water (The Nile) and tap water were spiked with 5, 20, and 100 ng/mL. Application of the method showed acceptable % recovery as shown in Table 4 and showed that the method could be applied in the analysis of repaglinide in water samples with high sensitivity and acceptable accuracy and precision. It should be noted that the method was validated in aqueous samples for the determination of repaglinide in environmental water.

**Table 4:** Application of vortex assisted-DLLME for determination of repaglinide in environmental water

River water			Tap water		
Spiked (ng/mL)	Found* (ng/mL)	%Recovery	Spiked (ng/mL)	Found* (ng/mL)	%Recovery
5	4.9	98.6	5	5.1	101.4
20	20.9	104.5	20	20.3	101.7
100	100.7	100.7	100	99.5	99.5
Mean		101.3			100.8
%RSD		2.95			1.17

^*^Mean of triplicate determination for each concentration

### Comparison of the proposed method and other reported methods

The sensitivity of the proposed vortex-assisted DLLME method was compared to different reported methods for the determination of repaglinide in different matrices in terms of LODs and LOQs (Table 5). The comparison shows that vortex-assisted DLLME can reach comparable levels of sensitivity with mass spectroscopy techniques, which is sophisticated, expensive, and requires highly expert technicians. Vortex-assisted DLLME does not require time, high consumption of organic solvents, or expensive and special cartridges such as liquid–liquid extraction (LLE) and solid phase extraction (SPE). In other words, vortex-assisted DLLME is a simple, rapid, cheap, and eco-friendly method.

**Table 5:** Comparison between the proposed method and other reported methods for repaglinide determination in different matrices

Sample	Sample preparation	Method	LOD (ng/mL)	LOQ (ng/mL)	Refs.
Human plasma	LLE	HPLC–UV	10	20	[2]
Human plasma	LLE	ESI-LC–MS	1	1	[47]
Human plasma	SPE	HPLC–UV	NR	20	[8]
Monkey plasma	Protein precipitation	LC–MS/MS	NR	1	[6]
Rabbit plasma	Protein precipitation	HPLC–UV	18	55	[48]
Human urine	-	UPLC-MS/MS	0.10	0.40	[7]
Human urine	Salting out	HILIC-MS/MS	NR	2	[10]
Urine	SPE	LC–MS/MS	5	NR	[11]
Tablet	Filtration and dilution	Ion pair HPLC–UV	27	81	[49]
Tablet	Filtration and dilution	HPLC–UV	NR	100	[50]
Tablet	Filtration and dilution	HPLC–UV	100	310	[51]
Tablet	Filtration and dilution	HPLC–UV	278	840	[52]
Tap water	vortex assisted-DLLME	HPLC–UV	0.40	1	This work

LLE: liquid–liquid extraction; SPE: solid-phase extraction; ESI: electrospray ionization; NR: not reported

## Conclusions

A vortex assisted-dispersive liquid–liquid microextraction coupled with HPLC/UV methodology was developed and validated for preconcentration and determination of repaglinide in environmental samples. The optimization of the experimental conditions was carried out using a factorial design model which allows studying the interaction between the various factors affecting the method efficiency in addition to saving time and effort. The response surface optimization provided a more efficient method for studying the variables simultaneously. The optimized conditions provided the highest possible enrichment factor. Vortex-assisted DLLME method offers a lot of advantages including a short time of analysis, simple procedures, and low consumption of organic solvents, which makes the method more economic and environmentally benign. The validation of the proposed method proved its sensitivity, accuracy, and reproducibility. The method was applied for environmental samples like river water and tap water providing reliable %recoveries. As well as it was applied to plasma and could be extended to be applied in the biological analysis of repaglinide.

## Supplementary Information

**Additional file 1:** Data of the model development, optimization and validation.

## Data Availability

Not applicable.

## Abbreviations

DLLME: Dispersive liquid–liquid microextraction
EF: Enrichment factor
FDS: Fraction of design space
