# Supplementary material for: Response surface optimization of a vortex-assisted dispersive liquid–liquid microextraction method for highly sensitive determination of repaglinide in environmental water by HPLC/UV
Source: BMC Chem. 2022 May 14;16(1):33. doi: 10.1186/s13065-022-00826-w (PMC9107645; doi:10.1186/s13065-022-00826-w)
Supplement: Supplementary file 1 — Additional file 1: Data of the model development, optimization and validation. [file 13065_2022_826_MOESM1_ESM.docx]

**Response surface optimization of a vortex-assisted dispersive liquid-liquid microextraction method for highly sensitive determination of repaglinide in Nile water by HPLC/UV**

Amira H. Kamal^a^, Mohamed A. Hammad^b^, Reham E. Kannouma^b^, Fotouh R. Mansour^a,c^

*^a^Department of Pharmaceutical Analytical Chemistry, Faculty of Pharmacy, Tanta University, Egypt, 31111*

*^b^Department of Analytical Chemistry, Faculty of Pharmacy, University of Sadat City, Egypt, 32958*

*^c^Pharmaceutical Services Center, Faculty of Pharmacy, Tanta University, Egypt, 31111*

**Supplementary Materials**

**Fig. S1:** Preliminary optimization of extractant type. Microextraction conditions: extractant volume: 50μL, disperser type: acetonitrile, disperser volume: 100μL, vortex time: 1 min, centrifugation time: 5min

**Fig. S2:** Preliminary optimization of disperser type. Microextraction conditions: extractant type: decanol, extractant volume: 50μL, disperser volume: 100μL, vortex time: 1 min, centrifugation time: 5min.

**Fig. S3:** Preliminary optimization of disperser volume. Microextraction conditions: extractant type: decanol, extractant volume: 50μL, disperser type: acetonitrile, vortex time: 1 min, centrifugation time: 5min.

**
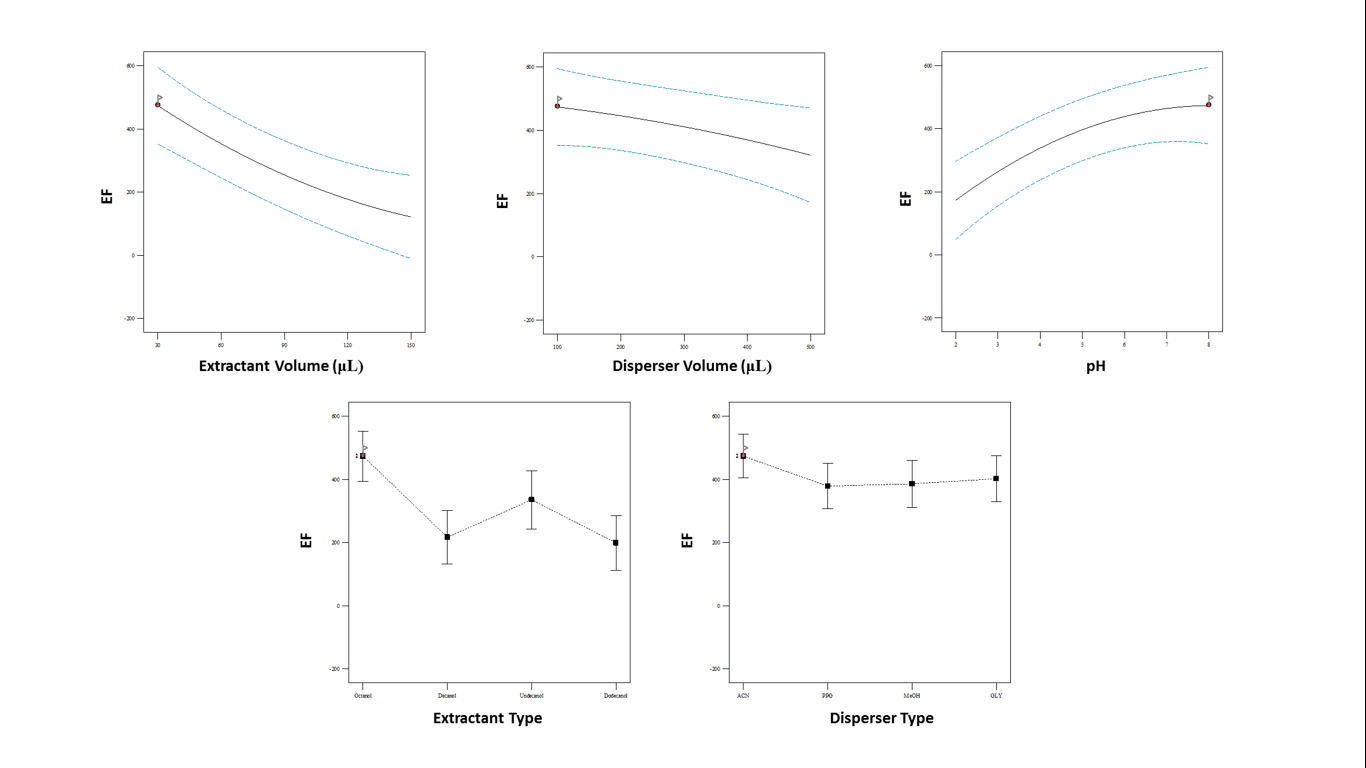
**

**Fig. S4:** Effect of different experimental variables on the enrichment factor.


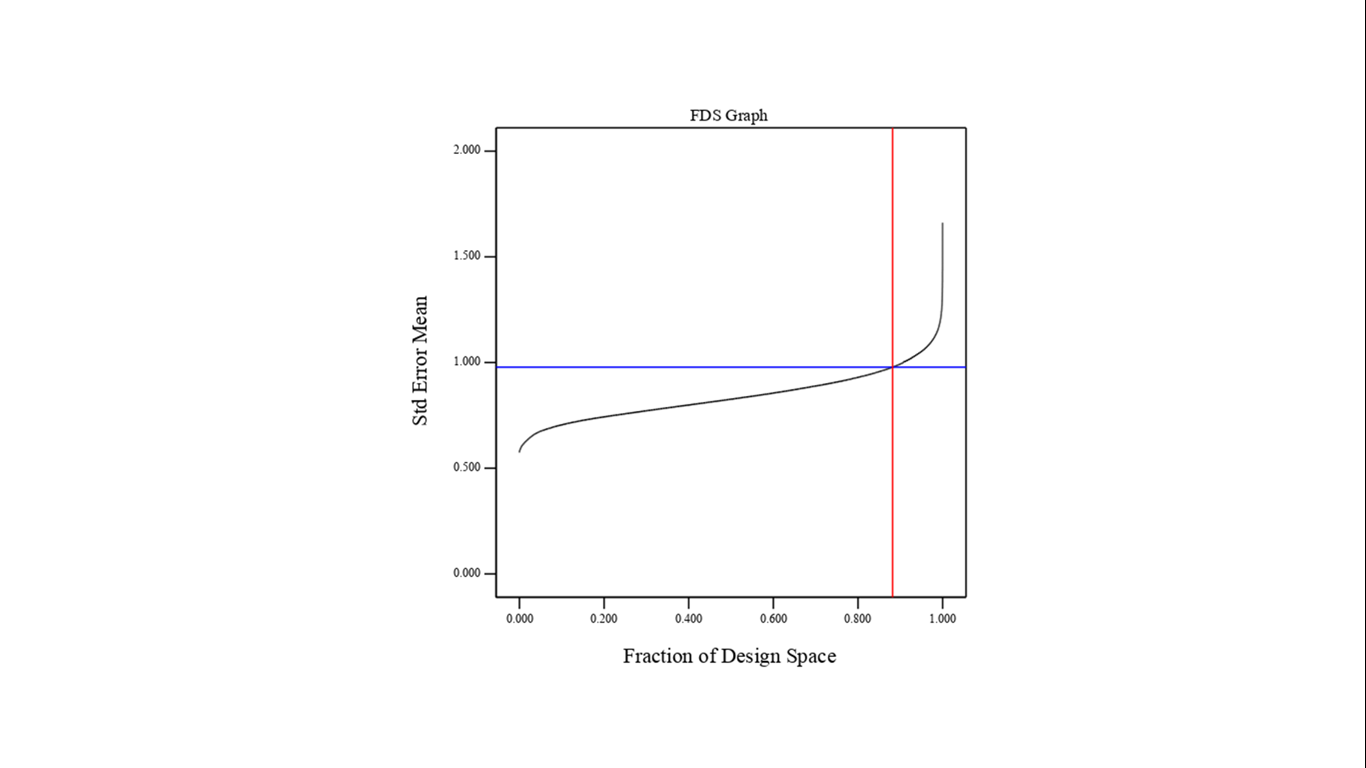


**Fig. S5:** Fraction of design space for the developed model.


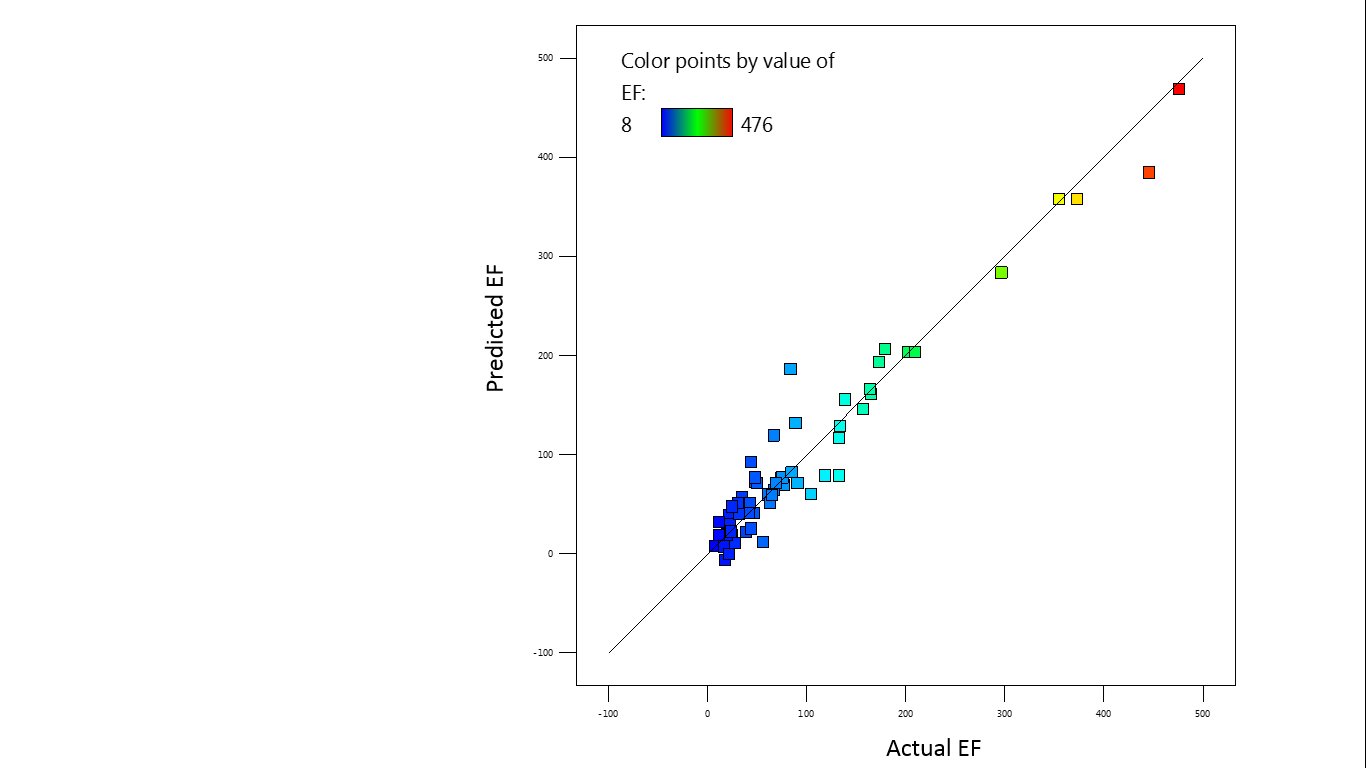


**Fig. S6**: The actual versus the predicted enrichment factors for the developed model.


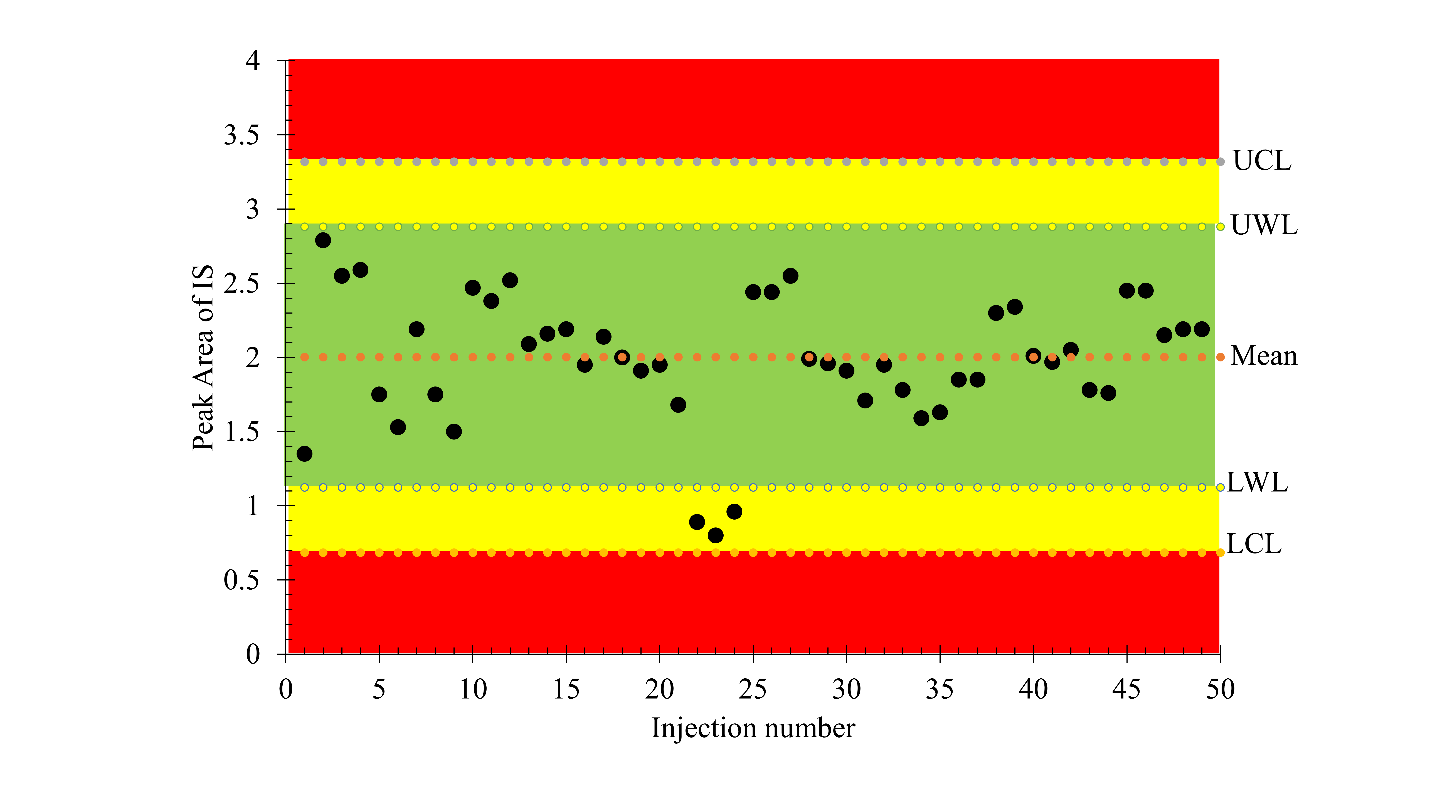


**(b)**

**(a)**


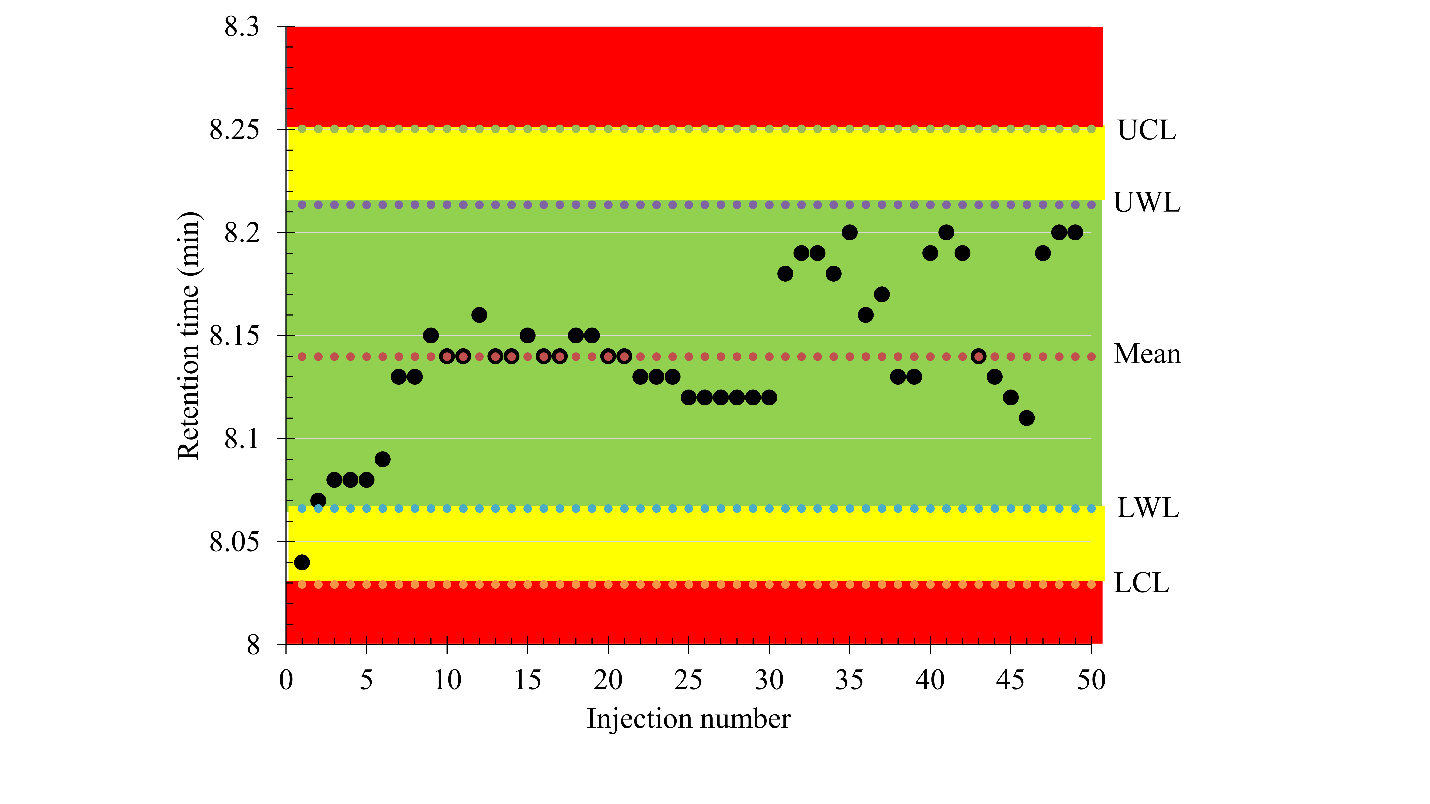


**Fig. S7:** Area under curve of internal standard (a) and retention times of repaglinide (b) from 50 consecutive runs. UCL: upper control limit, UWL: upper warning limit, LWL: lower warning limit, and LCL: lower control limit. The warning limits are ±2SD while the control limits are ±3SD

**Table S1** The 58 runs suggested with the aid of Design Expert 8^®^ software.

| **Day** | **Run** | **Extractant volume (μL)** | **Disperser volume (μL)** | **pH** | **Extractant type** | **Disperser type** |
| --- | --- | --- | --- | --- | --- | --- |
| Day 1 | 1 | 150 | 500 | 4.73 | Decanol | GLY |
| Day 1 | 2 | 73 | 280 | 8.00 | Undecanol | MeOH |
| Day 1 | 3 | 30 | 420 | 2.00 | Undecanol | ACN |
| Day 1 | 4 | 30 | 500 | 8.00 | Dodecanol | MeOH |
| Day 1 | 5 | 99 | 340 | 2.00 | Undecanol | PPG |
| Day 1 | 6 | 30 | 500 | 8.00 | Octanol | PPG |
| Day 1 | 7 | 150 | 100 | 2.00 | Octanol | MeOH |
| Day 1 | 8 | 150 | 100 | 8.00 | Decanol | ACN |
| Day 1 | 9 | 30 | 110 | 2.28 | Octanol | GLY |
| Day 1 | 10 | 150 | 500 | 4.73 | Decanol | GLY |
| Day 1 | 11 | 88 | 500 | 8.00 | Undecanol | GLY |
| Day 1 | 12 | 150 | 500 | 2.00 | Octanol | PPG |
| Day 1 | 13 | 30 | 100 | 2.00 | Undecanol | MeOH |
| Day 1 | 14 | 150 | 500 | 2.00 | Dodecanol | ACN |
| Day 1 | 15 | 150 | 445 | 8.00 | Decanol | MeOH |
| Day 1 | 16 | 35 | 100 | 8.00 | Octanol | ACN |
| Day 1 | 17 | 87 | 100 | 2.00 | Decanol | GLY |
| Day 1 | 18 | 30 | 100 | 2.00 | Dodecanol | ACN |
| Day 1 | 19 | 65 | 305 | 4.79 | Decanol | PPG |
| Day 2 | 20 | 67 | 100 | 7.49 | Dodecanol | GLY |
| Day 2 | 21 | 90 | 110 | 4.91 | Octanol | PPG |
| Day 2 | 22 | 59 | 390 | 3.29 | Octanol | GLY |
| Day 2 | 23 | 59 | 390 | 3.29 | Octanol | GLY |
| Day 2 | 24 | 30 | 500 | 2.00 | Decanol | MeOH |
| Day 2 | 25 | 117 | 500 | 7.25 | Octanol | MeOH |
| Day 2 | 26 | 90 | 110 | 4.91 | Octanol | PPG |
| Day 2 | 27 | 150 | 250 | 4.43 | Decanol | PPG |
| Day 2 | 28 | 150 | 305 | 2.00 | Undecanol | GLY |
| Day 2 | 29 | 150 | 105 | 8.00 | Undecanol | PPG |
| Day 2 | 30 | 36 | 450 | 3.11 | Dodecanol | PPG |
| Day 2 | 31 | 82 | 500 | 8.00 | Decanol | PPG |
| Day 2 | 32 | 30 | 295 | 8.00 | Decanol | GLY |
| Day 2 | 33 | 78 | 340 | 7.67 | Dodecanol | ACN |
| Day 2 | 34 | 150 | 100 | 2.00 | Dodecanol | PPG |
| Day 2 | 35 | 78 | 340 | 7.67 | Dodecanol | ACN |
| Day 2 | 36 | 75 | 100 | 5.15 | Decanol | MeOH |
| Day 2 | 37 | 150 | 285 | 5.60 | Octanol | ACN |
| Day 2 | 38 | 150 | 325 | 5.03 | Dodecanol | GLY |
| Day 2 | 39 | 127 | 320 | 2.00 | Decanol | ACN |
| Day 2 | 40 | 100 | 500 | 3.14 | Octanol | MeOH |
| Day 3 | 41 | 110 | 100 | 4.73 | Undecanol | ACN |
| Day 3 | 42 | 150 | 500 | 8.00 | Dodecanol | PPG |
| Day 3 | 43 | 95.5 | 340 | 2.00 | Dodecanol | MeOH |
| Day 3 | 44 | 30 | 500 | 2.00 | Dodecanol | GLY |
| Day 3 | 45 | 30 | 215 | 4.64 | Octanol | MeOH |
| Day 3 | 46 | 39 | 500 | 2.00 | Octanol | ACN |
| Day 3 | 47 | 30 | 100 | 2.00 | Decanol | PPG |
| Day 3 | 48 | 30 | 500 | 6.95 | Undecanol | PPG |
| Day 3 | 49 | 120 | 230 | 6.77 | Dodecanol | PPG |
| Day 3 | 50 | 30 | 215 | 4.64 | Octanol | MeOH |
| Day 3 | 51 | 150 | 100 | 8.00 | Octanol | GLY |
| Day 3 | 52 | 30 | 100 | 5.15 | Undecanol | GLY |
| Day 3 | 53 | 150 | 100 | 8.00 | Dodecanol | MeOH |
| Day 3 | 54 | 30 | 100 | 8.00 | Dodecanol | PPG |
| Day 3 | 55 | 150 | 500 | 8.00 | Undecanol | ACN |
| Day 3 | 56 | 31.5 | 465 | 5.84 | Decanol | ACN |
| Day 3 | 57 | 87.5 | 165 | 2.00 | Octanol | ACN |
| Day 3 | 58 | 150 | 500 | 4.04 | Undecanol | MeOH |
